# COVID-19 pandemic and the consequential effect on patients with endometriosis

**DOI:** 10.1093/hropen/hoac013

**Published:** 2022-03-18

**Authors:** Matilda Shaked Ashkenazi, Ole Linvåg Huseby, Gard Kroken, Adrian Soto-Mota, Marius Pents, Alessandra Loschiavo, Roksana Lewandowska, Grace Tran, Sebastian Kwiatkowski

**Affiliations:** Pomeranian Medical University, Szczecin, Poland; Pomeranian Medical University, Szczecin, Poland; Norwegian Joint Registry, Bergen, Norway; Metabolic Diseases Research Unit of the National Institute of Medical Sciences and Nutrition Salvador Zubirán, Mexico City, Mexico; Pomeranian Medical University, Szczecin, Poland; Università degli Studi della Campania Luigi Vanvitelli, Caserta CE, Italy; Pomeranian Medical University, Szczecin, Poland; Center for Criminology and Sociolegal Studies, University of Toronto, Toronto, Canada; Pomeranian Medical University, Szczecin, Poland

**Keywords:** endometriosis, COVID-19, questionnaire, quality of life, mental health, infertility, pelvic pain, healthcare systems, women’s health, coronavirus disease 2019

## Abstract

**STUDY QUESTION:**

What was the effect of the coronavirus disease 2019 (COVID-19) pandemic on healthcare and quality of life in those suffering from endometriosis?

**SUMMARY ANSWER:**

Our study reveals a clear correlation between the deterioration of the reported physical and mental state and impaired medical care for patients suffering from endometriosis during the COVID-19 pandemic.

**WHAT IS KNOWN ALREADY:**

The quality of life of patients suffering from endometriosis is compromised in a variety of aspects. In response to the ongoing COVID-19 pandemic, self-isolation practices aimed at curbing the spread of COVID-19 have severely complicated the availability of proper medical care worldwide.

**STUDY DESIGN, SIZE, DURATION:**

The study involved a cross-sectional international self-reported online survey. Responses were accepted between November 2020 and January 2021. The survey was prepared by the Department of Obstetrics and Gynaecology in a medical university setting. The survey contained 17 questions and was placed online. Cooperation with different endometriosis organizations around the world enabled distribution of the survey through their social media platforms.

**PARTICIPANTS/MATERIALS, SETTING, METHODS:**

The study participants (n = 3024 replies) originated from 59 countries. The questionnaire was created after a literature review and is partially based on the validated quality of life questionnaires, adjusted to the study question. The survey was then translated to 15 other languages following World Health Organization recommendations as closely as possible. Chi-square tests for independence were carried out for the analysis of the two variables: suspension of health services, and the patients mental and physical well-being.

**MAIN RESULTS AND THE ROLE OF CHANCE:**

Out of 3024 participants from 59 countries who submitted the questionnaire between November 2020 and January 2021, 2964 (98.01%) provided information that enabled a full analysis. For the 1174 participants who had their medical appointments cancelled, 43.7% (n = 513) reported that their symptoms had been aggravated, and 49.3% (n = 579) reported that their mental state had worsened.

In comparison, of the 1180 participants who kept their appointments, only 29.4% (n = 347) stated that their symptoms had been aggravated, and 27.5% (n = 325) stated their mental health had worsened. The results showed that there was a significant link between the reported deterioration of mental and physical wellbeing and impaired medical care (cancellation) (*P* ≪ 0.001). A total of 610 participants did not have medical appointments scheduled, and these participants followed a similar pattern as the participants who kept their appointments, with 29.0% (n = 177) reporting aggravation of symptoms and 28.2% (n = 172) reporting that their mental state had worsened.

**LIMITATIONS, REASONS FOR CAUTION:**

Cultural differences among international participants are to be expected and this may have affected how participants from different countries interpreted and answered the questionnaire. Translating the questionnaire into 15 different languages, even though incorporating backwards translation, could possibly lead to different interpretations of given questions, simply based on different wording in the languages. The majority of respondents (around 90%) were from Europe and South America and therefore the findings may not be generalizable to other locations.

**WIDER IMPLICATIONS OF THE FINDINGS:**

Further research is needed to assess the true impact and long-term consequences of the COVID-19 pandemic for patients living with endometriosis.

**STUDY FUNDING/COMPETING INTEREST(S):**

This study received no funding and the authors declare they have no relevant conflicts of interest.

**TRIAL REGISTRATION NUMBER:**

N/A.

WHAT DOES THIS MEAN FOR PATIENTS?

Current studies have highlighted how endometriosis usually has a negative effect on a woman’s quality of life; consequently, this study examined the effect of the coronavirus disease 2019 pandemic on patients suffering from endometriosis. Since the global pandemic has compromised access to healthcare systems and services, many women with endometriosis did not receive the standard care for their disease.

This research involved an online survey that was available internationally during the global pandemic. The survey included 17 questions regarding the general well-being, both physical and mental, of participants with endometriosis. It also included questions regarding the cancellation of planned appointments such as consultations and treatments. The results showed that there was a significant link between the reported deterioration of mental and physical wellbeing and impaired medical care. Cancellation of planned surgeries had the strongest negative effect on women’s lives.

This study demonstrates the importance of proper standard medical care for patients with endometriosis and emphasizes the need for appropriate preparation for similar circumstances in the future.

## Introduction

The existing literature demonstrates that the quality of life of women suffering from endometriosis was impaired in a multitude of ways, even before the coronavirus disease 2019 (COVID-19) pandemic (Moradi *et al.*, 2014; Arion *et al.*, 2020). These include reduced work productivity (De Graaff *et al.*, 2013) as well as negative effects on relationships, education and general well-being (Soliman *et al.*, 2017; van Poll *et al.*, 2020). Although numerous studies on the quality of life of patients suffering from endometriosis have been undertaken, many of them have a relatively small sample size (De Graaff *et al.*, 2013; González-Echevarría *et al.*, 2019; Della Corte *et al.*, 2020).

The rapid spread of COVID-19 around the globe has triggered dramatic and often transformational effects on routine health care practices (Birkmeyer *et al.*, 2020). COVID-19-related policies and recommendations have further reduced the availability of caregivers and compromised healthcare for patients suffering from a variety of conditions (Wallis *et al.*, 2020). In particular, the practice of obstetrics and gynaecology has been compromised across multiple countries (Eurofound, 2021).

Many medical centres have temporarily ceased offering surgical management for endometriosis, which is a crucial part of the management of the condition (Parasar *et al.*, 2017), and appointments for outpatient settings are currently being postponed or cancelled (OECD/European Union, 2020). These factors negatively impact the standard of care for these patients.

Additionally, the International Society of Ultrasound in Obstetrics and Gynecology has recommended postponing ultrasound evaluation of non-acute pelvic pain (Bourne *et al.*, 2020).

Furthermore, patients with endometriosis have reported their concerns with seeking medical help because of the fear of becoming infected with severe acute respiratory syndrome coronavirus 2 (SARS-CoV-2), the virus that causes COVID-19, in medical centres (Leonardi *et al.*, 2020a). Consequently, the quality of life of these patients has been drastically impaired by pain, subfertility, frustration about disease recurrence and uncertainty regarding the therapeutic options available to them (Ammar *et al.*, 2020; Pfefferbaum and North, 2020). These restrictions were reported to put patients with endometriosis at risk of negative psychological effects, in addition to those inflicted by mandated self-isolation (Gordon and Balsom, 2020).

This study aimed to explore the effect of the global COVID-19 pandemic on patients suffering from endometriosis across multiple countries, and to investigate the different approaches to the medical management of these patients based on their self-reported experiences.

## Materials and methods

This study employed an international cross-sectional survey, created along an initial qualitative phase that consisted of a scoping literature review. A computerized search of PubMed Central-US National Library of Medicine (PMC), Biomed Central Women Health (BMC) and Health Affairs resources was performed to identify registered articles about endometriosis and obstetrics and gynaecology management published before and during the current global pandemic, as well as registered articles regarding COVID-19 and the healthcare management. The search was conducted using the following terms: ‘Endometriosis and quality of life’; ‘endometriosis and COVID-19’; and ‘Healthcare and COVID-19’.

The literature review included comparative studies, qualitative studies, clinical trials, controlled and randomized controlled trials and multicentre studies. Several articles were selected on the basis of the following inclusion criteria: articles published in the last 5 years, articles published in English in peer-reviewed journals and questionnaire studies on endometriosis that consisted of self-reported surveys and cross-references checked. As the qualitative phase yielded analysis that was not essential for interpreting the questionnaire data, it is thus not discussed in this study. After finalizing the initial English survey, translations of the survey into 15 languages were initiated: Arabic, Farsi, Finnish, French, German, Greek, Hebrew, Italian, Norwegian, Polish, Portuguese, Russian, Spanish, Swedish and Turkish.

Translations were aimed at the conceptual equivalent of relevant phrases and words, as recommended by the World Health Organization criteria (World Health Organization, 2017), yet avoided a ‘word-for-word’ or literal translation. It aimed for all three phases of forward translation, expert panel and back translation for every language.

### Questionnaire structure

The questionnaire comprised four distinct sections. The first section collected basic information about the respondents, including their age, nationality and country of residence during the global pandemic. This information has not violated their anonymity; rather, it has enabled a categorization of the responses based on these details for a later evaluation.

The second section of the questionnaire was based on a review of Endometriosis Health Profile-30 (Khong *et al.*, 2010), a validated tool designed to measure the health-related quality of life in women with endometriosis (Bourdel *et al.*, 2019; Moradi *et al.*, 2019; Weeks, 2020). This section inquired about general patient- and disease-specific characteristics in order to determine the current specific condition that the respondent is diagnosed with, in addition to when they were diagnosed, the effect of endometriosis on their life, and how it might limit their activity. This section also included questions regarding the respondent’s current treatments, including fertility treatments.

The third section of the questionnaire investigated the effects of the global pandemic on respondents, incorporating ‘yes/no’ questions. Where it was appropriate, an option of ‘this is irrelevant for me’ was added to some of the questions. This section investigated whether the respondent had experienced any cancellations/postponement of appointments that were initially scheduled for the diagnosis, treatment or both of their endometriosis and/or infertility related to their endometriosis.

Responses to the fourth section of the questionnaire were measured on a numerical rating scale. The degree to which the respondents agreed with the statement given in each question was scored on a scale ranging from 1 to 5, with 1 representing ‘strongly agree’ and 5 representing ‘strongly disagree’. Each question incorporated statements regarding the effects of the pandemic on a respondent’s decision to seek medical help concerning their endometriosis condition, aggravation of symptoms owing to the current global situation, and changes in the respondent’s mental state, as well as statements concerning the medical management of their disease during the pandemic. Since the fourth section of the questionnaire contained a Likert scale, it was validated prior to publishing the questionnaire, through Cronbach Alpha (CA = 0.832. 95% CIs were calculated via Bootstrap as: 0.82–0.84).

The questionnaire was converted into an online self-administered survey, which was distributed using the social media channels provided by the cooperating organizations.

The web-based questionnaire allowed all participants who reported suffering from endometriosis to anonymously answer it, independent of their nationality. As most national organizations do not limit their content to other nationalities, it is fair to assume that participants of other nationalities had access to the questionnaire via engagement with these organizations, especially if they manage their content in a language that participants from these countries speak.

Participants filled out the survey on a voluntary basis with no monetary compensation or incentive to do so. The study did not employ pre-existing clinical databases of patient history or patient details. Participants were thus not recruited at healthcare centres, as is appropriate during a global pandemic.

Data were collected from these online self-administered surveys, and subsequently interpreted for further analysis. Information regarding cooperating centres and organizations which contributed to this research can be found in Supplementary Data 1.

### Ethical approval

The bioethics committee of the Pomeranian Medical University in Szczecin provided an exemption from an ethical consent-case number: KB-0012/34/03/2021/Z. Additionally, this study was also granted an ethical approval from the Turkish Ministry of Health: 2021-01-13T17_02_26, Başvuru Formu için tıklayınız//KONU No: KAEK/2021.01.27.

In accordance with the Polish law and in compliance with the Declaration of Helsinki principles for ethical conduct research, one can receive either a certificate of exemption of opinion (equivalent to an approval to conduct the research) or an opinion (with approval or rejection statement) if the research study involves human trial, personal identifiers or biological samples handling. As this study did not involve any of the above, it received a certificate of exemption of opinion (considered to be the equivalent of an approval statement). Moreover, the Ethics Committee that approved the conduct of this research did not advise seeking consent or approval from other committees—a process that is required when the committee deems it necessary. Instead, it found that it was sufficient to secure approval from the official board of the research institution of the principal investigator alone. One exception to this jurisdiction is Turkey, which is why independent Ethics approval was sought in Turkey. The Turkish Ministry of Health required approval from the ministry as well as ethical committee approval from the local principal investigator in any kind of research that includes COVID-19 information; thus, the research team obtained ethical approval from Turkey in order to allow the Turkish centre cooperating with it to distribute the questionnaire.

### Data analysis

Data analysis was conducted using R Core Team (2021). Chi-square tests for independence were carried out for the analysis of the two variables: suspension of health services, and the patients' mental and physical well-being.

## Results

Out of 3024 participants from 59 countries who submitted the questionnaire between November 2020 and January 2021, 2964 (98.01%) provided information that enabled proper analysis of the results. Table I shows the demographic and clinical characteristics of the participants. As described, the mean age of the participants is 33.2 (SD: ±7.5) and the distribution between the stages of endometriosis is as follows: Stage 1: 4.8% (n = 142); Stage 2: 9% (n = 267); Stage 3: 14.7% (n = 435); and Stage 4: 30.7% (n = 910). In total, 40.8% (n = 1210) of participants stated that they were not currently diagnosed with a specific stage of the disease.

**Table I:** Demographic and clinical characteristics of women with endometriosis who completed the survey.

Variable		N/mean	SD/%	Min–max	CI 95%
Age (years)		33.2	7.5	12–72	32.9–33.5
Age at diagnosis (years)		27.7	–	–	–
Endometriosis stage	Stage 1	142	4.8%	–	–
	Stage 2	267	9%	–	–
	Stage 3	435	14.7%	–	–
	Stage 4	910	30.7%	–	–
	Unknown*	1210	40.8%	–	–
How often do you seek treatment?	Once a year	668	22.5%	–	–
	Once every 6 months	1112	37.5%	–	–
	Multiple times during a half year	762	25.7%	–	–
	Multiple times a month	103	3.5%	–	–
	Other	319	10.8%	–	–
Patients reporting difficulties to conceive	Fertility problems: Yes	861	29%	–	–
	Treated	397	46.1%	–	–
	Untreated	464	53.9%	–	–
Effect of endometriosis on everyday life**	Severe limitation	1393	–	–	–
	Limits physical activity	1202	–	–	–
	Limits periodically	1574	–	–	–
	Does not limit	230	–	–	–
SARS-CoV-2 status***	Positive	183	6.2%	–	–
	Negative/not tested	2781	93.8%	–	–
Demographic distribution:					

	**Continent**	**Residence**		**Origin/nationality**	
		**N/mean**	**SD/percentage**	**N/mean**	**SD/percentage**
	Africa	24	0.8%	22	0.7%
	Asia	227	7.7%	231	7.8%
	Eastern Europe	156	5.3%	180	6.1%
	North America	74	2.5%	78	2.6%
	Northern Europe	432	14.6%	420	14.2%
	Oceania	86	2.9%	75	2.5%
	South America	331	11.2%	343	11.6%
	Southern Europe	1033	34.9%	1030	34.8%
	Western Europe	601	20.3%	585	19.7%

* Participants were not diagnosed with a specific stage or did not know their stage at the time when they completed the questionnaire.

** There are no percentages for the effect as the patients could choose multiple answers.

*** Participants reporting a positive SARS-CoV-2 test before or during answering the questionnaire.

SARS-CoV-2, severe acute respiratory syndrome coronavirus 2.

Table I also outlines the general and transformational effects that endometriosis imposes on the everyday life of participants. Only 230 participants stated that their condition has no significant effect or no effect at all on their everyday activities, while 1393 participants stated that they experience a severe compromise in their routine activities, such as going to work and housekeeping chores. Table I also summarizes the frequency with which the participants seek medical attention concerning their endometriosis, their fertility status and those who were previously or currently diagnosed with COVID-19.

**Figure 1.**
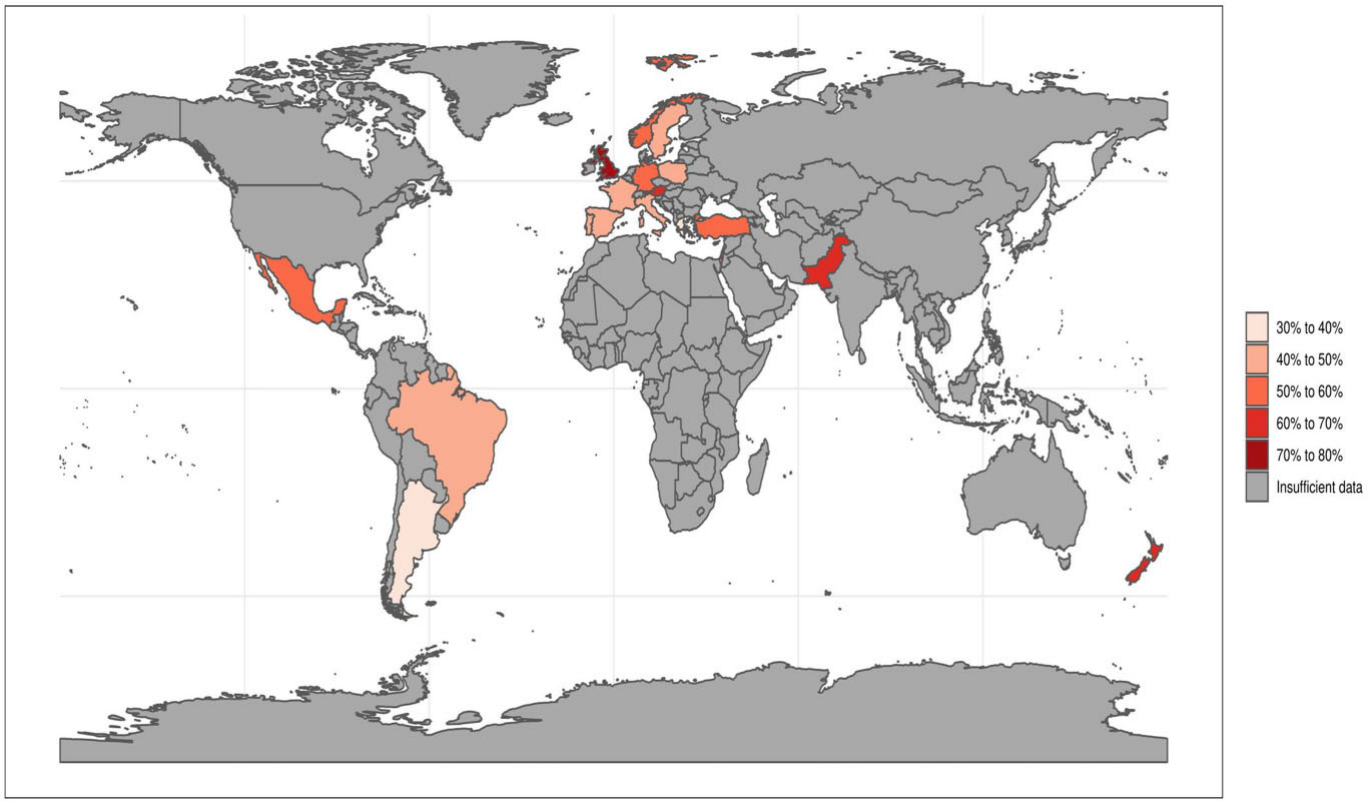
**Reported distribution of worsening of mental and physical well-being during the pandemic.** Heat map showing the percentage of participants reporting a worsening of both mental and physical health during the pandemic. The countries where participants reported from are shaded. The darker the shade, the higher the percentage of participants reporting worsening in both categories.

Table II shows the reported mental health changes that the participants experienced during the COVID-19 pandemic at the time of completing the questionnaire. Figure 1 outlines the demographic distribution of the participants, related to their reported worsening of mental and physical well-being.

**Table II:** Mental health changes experienced by participants during the coronavirus disease 2019 pandemic, at the time of completing the questionnaire.

		Participants reporting mental state deterioration			
		Yes	No	%***	*P*-values
		1076	1888	36.30%	
	**Cancellation/postponement:**				
Medical appointments	Yes	579	595	49.32%	≪0.001
	No	325	855	27.54%	
	Other*	172	438	28.20%	
Fertility treatment	Yes	116	139	45.49%	≪0.001
	No	178	417	29.92%	
	Other*	782	1332	36.99%	
Surgical appointments	Yes	227	168	57.47%	≪0.001
	No	329	676	32.74%	
	Other*	520	1044	33.25%	
SARS-CoV-2 status**	Positive	71	112	38.80%	0.5
	Negative/not tested	1005	1776	36.14%	

* Participants reporting to not hold appointments of the kind shown above, not used in chi squared test.

** Participants reporting a positive SARS-CoV-2 test before or during answering the questionnaire.

*** Percentage of people saying yes in the relevant category.

SARS-CoV-2, severe acute respiratory syndrome coronavirus 2.

Table II shows 36.3% of the participants reported that their mental health had worsened during the pandemic. A total of 1174 participants reported some kind of cancellation to medical appointments, and 49.3% of them stated that their mental health had deteriorated. In comparison, of the 1180 participants who did not experience cancellations and the 610 who did not have any scheduled appointments, 27.5% and 28.2% reported worse mental health, respectively. Table II also identifies the number of participants who reported that their scheduled fertility treatment and/or surgical appointments were postponed or cancelled. Among the participants reporting a worsening of mental health, 38.8% (n = 71) have tested positive for SARS-CoV-2, and 36.1% (n = 1005) have tested negative or were not tested at all.

Table III shows that 35.0% of participants feel their symptoms have been aggravated during the COVID-19 pandemic. For the 1174 participants who had their medical appointments cancelled, 43.7% reported that their symptoms had been aggravated. In comparison, from the 1180 participants who kept their appointments and the 610 that did not report to have any medical appointments scheduled, 29.4% and 29.0% stated that their symptoms had been aggravated, respectively. Table II also shows participants reporting that their scheduled fertility treatments and/or their surgical appointments were postponed or cancelled. Moreover, from the participants reporting symptomatic aggravation, 36.0% have tested positive for SARS-CoV-2, and 34.9% have tested negative or were not tested at all.

**Table III:** Data for participants who reported aggravated symptoms of endometriosis during the COVID-19 pandemic.

		Participants reporting aggravated symptoms			
		Yes	No	%***	*P*-values
		1037	1927	34.99%	
	**Cancellation/postponement:**				
Medical appointments	Yes	513	661	43.70%	≪0.001
	No	347	833	29.41%	
	Other*	177	433	29.02%	
Fertility treatment	Yes	104	151	40.78%	≪0.001
	No	157	438	26.39%	
	Other*	776	1338	36.71%	
Surgical appointments	Yes	207	188	52.41%	≪0.001
	No	338	667	33.63%	
	Other*	492	1072	31.46%	
SARS-CoV-2 status**	Positive	66	117	36.07%	0.8
	Negative/not tested	971	1810	34.92%	

* Participants reporting to not hold appointments of the kind shown above, not used for chi squared test.

** Participants reporting a positive SARS-CoV-2 test before or during answering the questionnaire.

*** Percentage of people saying yes in the relevant category.

COVID-19: coronavirus disease 2019; SARS-CoV-2, severe acute respiratory syndrome coronavirus 2.

Tables IV and V summarize the self-reported impact of the COVID-19 pandemic on medical healthcare and overall well-being. Most of the respondents (79%, n = 2354) had at least one healthcare appointment scheduled during the pandemic. Almost half (49.9%, n = 1174) of them reported at least one cancellation and almost 30% of the scheduled surgical and fertility treatments were cancelled (28.2%, n = 395 and 30.0%, n = 255 respectively). Additionally, almost half of the participants (48.7%, n = 959) reported they would have sought emergency gynaecological attention but refrained from doing so because of their fears concerning arriving at a medical institution at the time of the global pandemic.

**Table IV:** Respondents reporting impact of the coronavirus disease 2019 pandemic on medical healthcare.

	Yes n (%)	No n (%)	Does not apply n (%)	% cancelled appointments
Cancellations of any kind	1174 (39.6%)	1180 (39.8%)	610 (20.5%)	49.9
Cancelled surgeries	395 (13.3%)	1005 (33.9%)	1564 (52.72%)	28.2
Cancelled fertility treatments	255 (8.6%)	595 (20.1%)	2114 (71.3%)	30.0
Refrained from seeking emergency gynaecological attention	959 (32.3%)	1009 (34.0%)	996 (33.6%)	48.7

**Table V:** Respondents reporting impact of the coronavirus disease 2019 pandemic on medical healthcare and overall well-being.

	Strongly agree n (%)	Agree n (%)	Neither n (%)	Disagree n (%)	Strongly disagree n (%)	% Agree or strongly agree
Refrained from seeking any gynaecological attention	559	441	674	430	860	33.7
	(18.9%)	(14.9%)	(22.7%)	(14.5%)	(29.1%)	
Would seek more help without the pandemic	694	495	471	347	957	40.1
	(23.4%)	(16.7%)	(15.9%)	(11.7%)	(32.3%)	
Symptoms aggravated during the pandemic	599	438	650	381	896	35.0
	(20.2%)	(14.8%)	(21.9%)	(12.9%)	(30.2%)	
Mental state worsened during the pandemic	567	509	536	426	926	36.3
	(19.1%)	(17.2%)	(18.1%)	(14.4%)	(31.2%)	
Their condition would have been managed better without the pandemic	713	450	522	388	891	39.2
	(24.1%)	(15.2%)	(17.6%)	(13.1%)	(30.1%)	

More than one-third of the respondents reported that their symptoms or mental well-being deteriorated during the pandemic (35%, n = 1037 and 36%, n = 1076 respectively) and 39% (n = 1163) of them believed that their condition would have been managed better if the COVID-19 pandemic had not occurred. Conversely, 43% (n = 1279) of the respondents asserted that the ways in which they manage their endometriosis have not particularly changed because of or during the COVID-19 pandemic. In total, 18% (n = 522) of the participants reported neither an impairment of their endometriosis management, nor lack of change.

## Discussion

This study presents a view of a problem that was evident even before the pandemic, in which compromising the resources available to treat and diagnose endometriosis significantly affects the overall quality of life of those who suffer from it (Leonardi *et al.*, 2020b). The pandemic has amplified the existing compromises on the general resilience of healthcare systems worldwide, especially as they relate to the management of space, human and material resources (OECD/European Union, 2020).

Drawing on data from 2964 participants from 59 countries, who are diverse in terms of ethnicity, nationality, and socioeconomic status, provided the opportunity to present a well-established estimation accounting for the management of their disease. As expected, our data indicate that general absence of care directly impacts quality of life for patients suffering from endometriosis.

Our study detected important alterations in respondents’ mental and physical well-being, and almost 50% reported a decline in one or both during the COVID-19 pandemic. Our findings suggest that this reported decline in physical and mental well-being can be attributed to the cancellation/postponement of medical appointments, including surgical and fertility treatments. This is supported by other studies, which have reported considerable negative impacts on women’s mental health and quality of life while they await fertility treatment during the COVID-19 pandemic (Gordon and Balsom, 2020).

Similarly, 28% of women have had their scheduled surgical appointments delayed, which can postpone both the proper diagnosis and treatment. Reports are conflicting about the relevance of these delays in the healthcare of patients (Unger and Laufer, 2011; Hudelist *et al.*, 2012). Nonetheless, even in non-pandemic circumstances, several studies have reported a delay in diagnosis of 7–12 years in women with endometriosis (Hadfield *et al.*, 1996; Husby *et al.*, 2003; Ballard *et al.*, 2006; Staal *et al.*, 2016). Thus, it is fair to assume that an even longer delay in both the diagnosis and treatment can be expected during the COVID-19 pandemic. Additional follow-up is needed to determine the true impact that this delay will have.

Most procedures and appointments in endometriosis healthcare are elective. However, the fact that almost 40% of respondents believe that their condition would have been better managed were it not for the COVID-19 pandemic indisputably deserves attention. Importantly, more than one-third of the participants reported physical or mental harm that was attributable to the effects of the pandemic on their healthcare, and the consequences of these detriments are yet unknown. Long-term follow-up studies will also be needed to assess this.

Finally, it is concerning that almost half of the participants refrained from seeking emergency gynaecological attention. It remains possible that implementation of social restrictions will be required again in the future. Since all healthcare systems should be prepared to face future high-demand challenges, it is necessary to design and implement strategies to allow all non-COVID-19 emergencies to be properly managed.

With a lack of direct and easily quantifiable outcomes, it will be particularly difficult to estimate the consequences of the COVID-19 pandemic on people living with non-lethal, highly prevalent chronic diseases such as migraine, fibromyalgia and endometriosis.

Despite the inherent differences between these illnesses, it is likely that at least some of the repercussions for patients with endometriosis that were documented will be reflected in other diseases. Insight from this study should prove useful for updating endometriosis clinical management guidelines all around the world, and for improving the resilience of healthcare systems against future high-demand challenges.

Owing to a low number of respondents who tested positive for SARS-CoV-2, any statistical test of association would be underpowered, and the research team are therefore unable to say whether there is a significant connection or not.

With an international questionnaire, issues of cultural differences and subjective answers are likely inevitable. In most cases, the research team ensured that at least two people who spoke the target language were translating the survey from English to the target language. However, the research team could not always ensure that two translators, whose mother tongue was English, were also both fluent in the target language for the backward translation.

Further limitations with a multiple-choice questionnaire are that participants can allude to different meanings when selecting the same answer. This problem increases when trying to reach an international sample of people. Furthermore, the questionnaire was anonymous, and there is no confirmation of whether the participants have only responded once and whether they answered honestly, although there is little reason to suspect otherwise, given that there was no incentive to take this questionnaire. The same might apply for the diagnostic credibility of the participants. Although the context of the study did not allow verification of the self-reported histological data, there is no incentive for participants to misrepresent their answers.

Distribution of the survey solely online, through various platforms and with the help of national and international endometriosis organizations, resulted in varying levels of success, and in some countries, the research team did not manage to release the questionnaire at all. Europe and South America were more represented than other areas, with around 90% of the respondents residing in these continents.

As the main objective of the study was to obtain a worldwide picture of how the pandemic affected the healthcare of patients with endometriosis and their related experiences, it did not assess the differences between the healthcare systems around the globe. There is also reason to believe that owing to the continuously dynamic changes during the pandemic, it might have been difficult to precisely assess the relevance of these differences as they would change frequently during that period.

The intention of this paper is therefore not to focus on the differences between countries but on the general rather than the specific effects of absence of care. Considering the study’s statistical power, the findings are unlikely to be coincidental. While the global COVID-19 pandemic is ongoing, the present study’s findings are not limited to COVID-19 but enable us to understand the consequences of general absence of care in many forms and to eventually conclude how to better manage chronic diseases in the future, and in relation to endometriosis.

Further research is needed to assess the true impact and long-term consequences of the COVID-19 pandemic for patients living with endometriosis. For now, simpler measurements can be implemented to mitigate the detrimental effects that limited health care has had on the reported health of the participants. Telemedicine with video consultations shows promise for some patients (Grimes *et al.*, 2020). This cannot replace necessary face-to-face consultations and surgical procedures, but can perhaps help patients who have suboptimal treatment, as they can be followed up digitally. In our study, questions were aimed towards classical medical management, in the way participants were managed prior to the pandemic. Thus, questions did not include data concerning telemedicine or other similar forms of consultations that were later adopted in some of the countries in order to cope with the current situation. It is thus reasonable to assume that interventions such as these could change the answers of some of the participants. However, since these methods were adopted at very different time points throughout the pandemic, it was not easy to assess whether the possibility of interventions like these would yield significant differences in patient responses.

## Conclusion

There are multiple components affecting the quality of life of women suffering from endometriosis. Our study reveals a clear correlation between deterioration of the reported physical and mental state and impaired medical care for patients suffering from endometriosis during the COVID-19 pandemic. The largest difference in reported well-being was found among patients who were supposed to undergo surgical procedures but had their appointments cancelled or postponed because of the pandemic.

## Data availability

The data underlying this article will be shared on reasonable request to the corresponding author.

## Supplementary Material

Supplementary_Data_1

Supplementary_Data_2
