# Supplementary material for: COVID-19 pandemic and the consequential effect on patients with endometriosis
Source: Hum Reprod Open. 2022 Mar 18;2022(2):hoac013. doi: 10.1093/hropen/hoac013 (PMC8982367; doi:10.1093/hropen/hoac013)
Supplement: Supplementary_Data_1 [file hoac013_supplementary_data_1.docx]

**Supplementary Data 1**

**Centers and Organizations-**

**Centers:**

Cooperating centers contributed to this research by intellectual means in their field of expertise. The main research team consulted with them in order to enhance the quality of this study during its different stages.

The Department of Woman, Child, and General and Specialized Surgery- University of Campania Luigi Vanvitelli, Napoli, Italy: Consultation regarding the methodology of the study and the analysis of the results

Department of Hygiene and Epidemiology, Pomeranian Medical University in

Szczecin, Poland: Recommendations regarding the structure of the questionnaire and its validation

**Organizations:**

There was no incentive for the organizations below to cooperate with the research team.

The following organizations contributed to this research both through distribution of the questionnaire and by the means mentioned below:

World Endometriosis Organizations: Distribution of the questionnaire on social media and recommendation of relevant literature

Turkish endometriosis and Adenomyosis Society, Turkey (organization): Distribution of the questionnaire on social media and guidance during literature review

The organizations below collaborated by the means of circulating the survey on their respective social media platforms.

- Endometriose-Vereinigung Deutschland e.V (Germany).

- Association EndoFrance (France)

- ENDOmind Franc/ endofrance (France)

- Endometrioseforeningen Norge (Norway)

- Endometriosis Israel (Israel)

- Endometriosis New Zealand (New Zealand)

- Endometriosföreningen Sverige (Sweden)

- World Endometriosis Organization

- World Endometriosis Society
