# Supplementary material for: COVID-19 pandemic and the consequential effect on patients with endometriosis
Source: Hum Reprod Open. 2022 Mar 18;2022(2):hoac013. doi: 10.1093/hropen/hoac013 (PMC8982367; doi:10.1093/hropen/hoac013)
Supplement: Supplementary_Data_2 [file hoac013_supplementary_data_2.docx]

**Supplementary Data 2**

**Translation:**

**Arabic-** Sahl Challar, M.D.* (2023) Pomeranian Medical University in Szczecin

Mohammed Aljaff, M.D.* (2023) Pomeranian Medical University in Szczecin, Khalil Gharios, Business Administration, (class of 2020), Coventry University, UK.

**Finnish-** Aurora Henriksson, M.D.* (2023) Pomeranian Medical University in Szczecin, Elena Lekkas, M.D.* (2026) Pomeranian Medical University in Szczecin,

**French-** Mithula Shanmugasuntharam, M.D.* (2023) Pomeranian Medical University in Szczecin, Laura Besenbruch, Actress in Paris.

**German-** Isabella Kuhn, M.D.* (2023) Pomeranian Medical University in Szczecin,
Miriam Hehlmann, M. Sc., University of Trier in Germany

**Greek-** Roxani - Magdalini Sidirokastriti, MA (Translation Studies), University of Birmingham U.K, Marina Lemis, Law LLB University of Reading UK, JMW Solicitors LLP, Anna Diakou, Law LLB University of Reading UK, Slaughter and May

**Hebrew -** Shaked Ashkenazi, M.D.*  (2023) Pomeranian Medical University in Szczecin, Hadas Yeverechyahu, Tel-Aviv University

**Italian-** Alessandra Loschiavo**,** M.D* (2021/2022) Università degli Studi della Campania Luigi Vanvitelli, Marta Lo Presti, UniCredit Bank - Milan

**Norwegian-** Ole Linvåg Huseby, M.D* (2023) Pomeranian Medical University in Szczecin, Daniel Hoseth Nilsen, M.D. (2018) Pomeranian Medical University in Szczecin

**Persian**-Leila Amini, PhD, Nursing and amidwifery School, Iran University of Medical Sciences, Iran, Tahmine Salehi, Tahmine Salehi, B.S., M.S. in Nursing Management, PhD in nursing. Associate Professor

**Polish-** Roksana Lewandowska, M.D.* (2021), Pomeranian Medical University in Szczecin

**Portuguese-** Beatriz Pestana Figueira Santos Faria, M.D.* (2022), Faculdade de Medicina da Universidade de Lisboa

**Russian-** Smolkin Yaakov, M.D. (2017), Pomeranian Medical University in Szczecin, Nikol M. M, Kolin Zolotnitsky

**Spanish-** Pablo Ignacio Soto Mota, Ph.D. Research Scholar, Norwegian School of Economics, Juan Sebastián Garrigues Vega, M.D* (2024), Pomeranian Medical University in Szczecin

**Swedish-** Kwabena Owusu-Mari, M.D.* (2026), Pomeranian Medical University in Szczecin

**Turkish-** Şeyma Taştekin, M.D.* (2022), Trakya University School of Medicine in Turkey
